# Assessment of prenatal depression among U.S. pregnant women without access to paid sick leave and regular place of care: National Health Interview Survey of U.S.-born and non-U.S.-born

**DOI:** 10.1016/j.pmedr.2023.102322

**Published:** 2023-07-14

**Authors:** Saanie Sulley, David Adzrago, Lohuwa Mamudu, Emmanuel A. Odame, Paul H. Atandoh, Ishmael Tagoe, David Ruggieri, Lisa Kahle, Faustine Williams

**Affiliations:** a National Healthy Start Association, Washington, DC, USA; b Division of Intramural Research, National Institute on Minority Health and Health Disparities, National Institutes of Health, Bethesda, MD, USA; c Department of Public Health, California State University, Fullerton, CA, USA; d Department of Environmental Health Sciences, School of Public Health, University of Alabama at Birmingham, Birmingham, AL, USA; e Department of Statistics, Western Michigan University, Kalamazoo, MI, USA; f Division of Health Services, College of Nursing and Advanced Health Professions, The Chicago School of Professional Psychology, Chicago, IL, USA; g Information Management Services, Inc., Calverton, MD, USA

**Keywords:** Prenatal depression, Place of care, Paid sick leave, U.S.-born, Non-U.S.-born

## Abstract

Prenatal depression is one of the most common risks during pregnancy. This study examined the prevalence and likelihood of prenatal depression association with sociodemographic factors, paid sick leave, and place of care among U.S. pregnant women. We conducted bivariate Chi-square tests to assess the statistical difference and multivariable logistic regression models to assess the association of prenatal depression using the National Health Interview Survey, cross-sectional data from 2010 to 2019 of pregnant women aged 18–44 years (N = 957). The prevalence of prenatal depression was 40.6%, 28.5%, and 27.2% among White, Black, and other racial pregnant women, respectively. Pregnant women with no regular/routine place of care had a prenatal depression prevalence rate of 58.1%, and those without access to paid sick leave had 46.9%. Also, pregnant women without access to paid sick leave were found to have an increased likelihood of reporting prenatal depression ([adjusted odds ratio] AOR = 2.50, 95% CI = 1.72–3.64), as well as those without a regular place of care (AOR = 2.43, 95% CI = 1.32–4.47). The findings identify factors that need to be addressed to minimize depression among U.S. pregnant women and establish the need for tailored interventions to address prenatal depression.

## Highlights

• Pregnant women with depression are at increased risk of perinatal depression (PND).
• PND prevalence higher among pregnant women without routine place of care.
• Women without paid sick leave and routine place of care had higher odds of PND.
• Access to mental health services during pregnancy is necessary.

## 1 Introduction

An estimated 1 in 10 women in the United States (U.S.) suffers from depression (Zhou et al., 2019). However, prenatal depression and other mental disorders are higher during pregnancy and postpartum (Bauman et al., 2020, Langan and Goodbred, 2016). According to the American College of Obstetricians and Gynecologists, perinatal depression is one of the most common medical complications during pregnancy and postpartum (ACOG Committee, 2018). Several factors, including biological, environmental, and psychological changes or stressors, have been associated with a high prevalence of depression during pregnancy and postpartum (Corwin and Pajer, 2008, Yim et al., 2015, Yu et al., 2021). Other studies have also found that depression during pregnancy negatively impact infants' physical and cognitive development (Deave et al., 2008, O'Hara and McCabe, 2013, Plant et al., 2015, Urizar and Muñoz, 2022).

According to a study by Haight et al. (2019), the rate of depression during delivery hospitalization in the U.S. was 28.7 per 1000 in 2015 compared to 4.1 per 1000 hospitalizations in 2000. These findings from a nationally representative sample further support the notion of the increasing prevalence of depression among the pregnant population in the U.S. Depression complications directly or indirectly impact maternal and child health outcomes such as increased obstetric complications (Nelson et al., 2013) preterm labor (Dayan et al., 2002, Dayan et al., 2006, Dole et al., 2003), decreased breastfeeding likelihood (Borra et al., 2015, Ystrom, 2012), and inadequate nutrition for mother and newborn (Barker et al., 2013, Hurley et al., 2008).

Risk factors such as adverse childhood experiences (Racine et al., 2020), relationships, history of mental disorders, and socioeconomic status (SES) (Amuli et al., 2021, Dagher et al., 2021, Field, 2017, Lancaster et al., 2010) have also been associated with increased prenatal depression. Studies have found some associations between the lower presentation of depression among individuals with some level of social, emotional, and partner support (Cluxton-Keller and Bruce, 2018, Negron et al., 2013). These support dynamics are vital concerning the employment and earnings of mothers during pregnancy and after birth (Sandler and Szembrot, 2019). In addition, a review by Underwood et al. (2016), found that 39% of those that experienced antenatal depression also had postnatal depression. The findings above further support the need to understand the factors contributing to these risks and develop tailored strategies to address the underlining determinants of depression in the prenatal period.

According to a Pew research report, the percentage of women giving birth in the past year was higher among immigrants (i.e., 7.5%) than among U.S.-born (i.e., 5.7%) (Budiman, 2020). Several studies have also found differences in pregnancy outcomes with characteristics such as obesity, birth weight, and infant mortality (Amuli et al., 2021, Chen et al., 2009, Ely et al., 2020, Huo et al., 2021, Jacques et al., 2019) among the U.S.-women and non-U.S.-born populations (Green et al., 2021, Singh and Yu, 1996). These variances in maternal and infant health outcomes necessitate understanding factors contributing to depression during pregnancy among U.S.-born and non-U.S.-born. Additionally, there is limited research investigating the impact of the diverse sociodemographic factors, access to paid sick leave, and place of care that may affect or exacerbate prenatal depression among the U.S.-born and non-U.S.-born pregnant populations.

Socioeconomic status and health behaviors are important determinants of health. Previous studies have reported that low SES increases the risk of antenatal depression (Ana et al., 2021, Fairthorne et al., 2019, Verbeek et al., 2019). Another study that examined the level of physical activity among pregnant women and its effect on prenatal and postnatal depressive symptoms noted that women who engaged in low physical activity were more likely to report being depressed (Ana et al., 2021). Given the potential stressors associated with pregnancy and employment loss, it is crucial to understand the relationship between various socioeconomic and behavioral factors on prenatal depression in the U.S. It is also essential to examine how organizational or social policies such as access to paid sick leave and regular or routine place of care impact the psychological well-being of pregnant women among the subgroup populations within the U.S.-born and non-U.S.-born status, given the diversity in workplace dynamics among U.S populations. Such an understanding could aid stakeholders (i.e., employers, policymakers, and insurance companies) in developing strategies to address prenatal depression and improve the services and resources available to the pregnant population to mitigate depression during the prenatal period.

## 2 Methods

### 2.1 Study design and participants

**Fig. 1:**
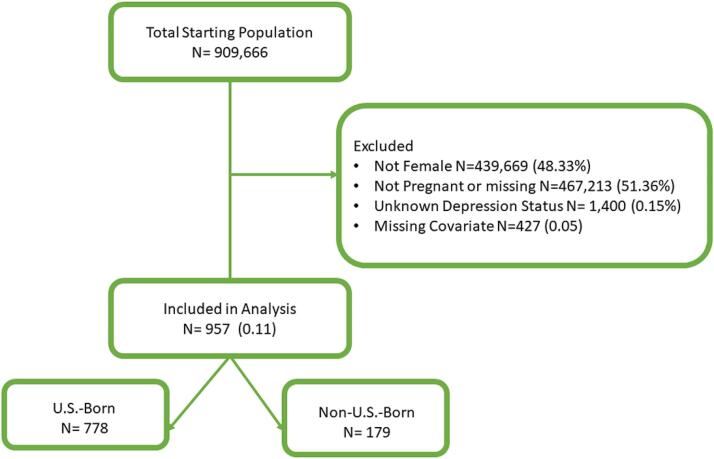
Consort diagram for Depression and Anxiety among U.S. Pregnant Population, NHIS 2010–2019.

Data for this study came from the National Health Interview Survey (NHIS), 2010–2019 (see https://www.cdc.gov/nchs/nhis/index.htm). The NHIS is a cross-sectional household interview survey targeting the civilian noninstitutionalized U.S. adult and child population. The analyses conducted in this study were restricted to U.S.-born and non-U.S.-born pregnant women aged 18–44 years with depression. Although individuals born in U.S. territories are citizens, they are not U.S.-born. Hence, we included them as non-U.S.-born. Participants with missing responses for any variables of interest were excluded from the analysis (i.e., 908,709) to minimize any statistical errors, resulting in a final sample of 957 pregnant women. See Fig. 1 for details. Since NHIS data has been de-identified and made publicly available, we did not require Institutional Review Board approval for the study.

### 2.2 Measures

The primary outcome of interest was self-reported prenatal depression status among pregnant women. Individuals were identified as depressed based on three questions: First, *“Thinking about the last time you felt depressed, how depressed did you feel?”* The response options were: *“1 = a little; 2 = a lot; 3 = somewhere in between; 4 = don’t know.”* These were recategorized as “Yes” (i.e.*, options 1, 2, and 3*) for depressed and “No.” (i.e.*, option 4*) as not depressed. The second question was *“How often do you feel depressed?”* Response options were: *“1 = daily; 2 = weekly; 3 = monthly; 4 = a few times a year; and 5 = never.”* Similar to the first question, we recategorized the response options *(*i.e.*, options 1, 2, 3, and 4) as “Yes”* for depressed *and “No”* for not depressed. *The third question was “Do you take medication for depression?”* with responses *“Yes”* and *“No.”* All *“Yes”* recategorized responses to questions 1 and 2 as well as question 3 were combined to indicate the presence of depression in pregnant women, and all *“No”* as not depressed.

The independent variables included in our analysis were sociodemographic (i.e., age, race), socioeconomic (i.e., class of work, employment status, the ratio of family income to poverty threshold, health insurance), health characteristics (i.e., body mass index (BMI), diabetes, hypertension, regular place of care), behavioral characteristics (i.e., smoking status, alcohol status, and leisure time physical activities), access to paid sick leave, and region of residence. Age was dichotomized into *“18*–*30”* and *“31*–*44*” years, while race was categorized into *“White,” “Black,”* and *“Other.”* Household/family size was combined into two groups of *“1*–*4”* and “≥*5”*. Family income levels were classified based on the ratio of family income to poverty guidelines. Class of work was categorized as *“Private”* and *“Government”* and employment status as *“Employed”* and *“Unemployed.”* Independent variables such as diabetes and hypertension status were classified as *“Yes,”* having the disease and *“No,”* not having the disease. Regular place of care was dichotomized as 0= *“No,”* not having a regular place of care and 1+ *“Yes,”* having a regular place of care. Further, access to paid sick leave status was categorized into “*Yes*” and “*No*.” Alcohol and smoking status were dichotomized into *“Never*” and *“Ever,”* whereas leisure-time physical activity as *“Inactive/Insufficient”* and *“Physically Active.”* Finally, individual’s region of residence was grouped into “*Northeast*,” “*North Central/Midwest*,” “*South*,” and “*West*.”

### 2.3 Statistical analysis

We estimated the prevalence of prenatal depression and conducted a bivariate analysis using a Chi-square test to ascertain the differences in prenatal depression between the various categories of sociodemographic (e.g., age, race), socioeconomic (e.g., health insurance, household size, family income level, employment status), health factors (e.g., BMI, regular place of care, hypertension, and diabetes status), behavior factors (e.g., alcohol, smoking, and leisure-time physical activity), paid sick leave, and region of residence. See Table 1 for details. Next, we conducted a multivariable logistic regression analysis to assess the association between the likelihood of experiencing prenatal depression and the above-stated independent factors, as shown in Table 2. All analyses were conducted using the Software for the Statistical Analysis of Correlated Data (SUDAAN) March 2018, Research Triangle Institute, North Carolina. Results are reported as frequencies, percentages, adjusted odds ratios (AORs), two-sided 95% confidence intervals (CIs), and statistical significance at p < 0.05. All the analyses were weighted using the NHIS sampling survey weight to provide nationally representative estimates and adjust for non-response biases.

**Table 1:** Bivariate Descriptive Characteristics and Prevalence of Prenatal Depression among Pregnant Women Stratified by Place of Birth, NHIS 2010–2019.

Characteristics	Total (Weighted %) [N = 957; 100%]			U.S.-Born (Weighted %) [N = 778; 81.3%]			Non-U.S.-Born (Weighted %) [N = 179; 18.7%]		
	Depressed [n = 368; 38.5%]	Not Depressed [n = 589; 61.5%]	P-value	Depressed [n = 340; 43.7%]	Not Depressed [n = 438; 56.3%]	P-value	Depressed [n = 58; 32.4%]	Not Depressed [n = 121; 67.4%]	P-value
Age Group			0.7718			0.2344			0.1466
18–30	228 (37.9)	364 (62.1)		196 (37.5)	318 (62.5)		32 (40.5)	46 (59.5)	
31–44	140 (39.0)	225 (61.0)		114 (42.8)	150 (57.2)		26 (27.4)	75 (72.6)	

Race			**0.0333**			0.1851			**0.0127**
White	296 (40.6)	441 (59.4)		250 (40.4)	365 (59.6)		46 (41.8)	76 (58.2)	
Black	46 (28.5)	94 (71.5)		43 (29.8)	82 (70.2)		3 (21.2)	12 (78.8)	
Other	26 (27.2)	54 (72.8)		17 (42.2)	21(57.8)		9 (14.6)	33 (85.4)	

Grouped BMI			**0.0049**			**0.0088**			0.5122
<18.5–24.9 (Underweight or Normal)	106 (29.9)	220 (70.1)		86 (30.0)	162 (70.0)		20 (29.6)	58 (70.4)	
25–29.9 (Overweight)	104 (37.9)	172 (62.1)		84 (39.2)	134 (60.8)		20 (32.5)	38 (67.5)	
≥30 (Obese)	158 (46.5)	197 (53.5)		140 (47.1)	172 (52.9)		18 (42.6)	25 (57.4)	

Household/Family Size			0.6004			0.6209			0.7764
1–4	316 (37.8)	520 (62.2)		26 8(38.7)	415 (61.3)		48 (33.3)	105 (66.7)	
≥5	52 (41.1)	69 (58.9)		42 (42.3)	53 (57.6)		10 (36.6)	16(63.4)	

Ratio of Family Income to Poverty Threshold			**0.0096**			**0.0309**			0.1558
<1.00	96 (49.5)	104 (50.5)		81 (49.5)	85 (50.5)		15 (49.7)	19 (50.3)	
≥1.00	272 (36.1)	485 (63.9)		229 (37.2)	383 (62.3)		43 (30.3)	102 (69.7)	

Health Insurance			0.5988			0.5457			0.7277
No	32 (42.2)	37 (57.8)		19 (45.0)	19 (55.0)		13 (37.3)	18 (62.7)	
Yes	336 (38.0)	552(62.0)		291 (38.8)	449 (61.2)		45 (33.2)	103 (66.8)	

Class of Work			0.9910			0.6192			0.3679
Private	319 (38.3)	501(61.7)		267 (39.6)	390(60.4)		52 (31.9)	111 (68.1)	
Government	49 (38.3)	88 (61.4)		43 (36.6)	78(63.4)		6 (50.3)	10 (49.7)	

Employment Status			0.2916			0.2685			0.7772
Employed	215 (36.7)	390 (63.3)		186 (37.4)	320 (62.6)		29 (32.7)	70 (67.3)	
Unemployed	153 (40.9)	199 (59.1)		124 (42.4)	148 (57.6)		29 (35.3)	51 (64.7)	

Diabetes			0.1686			0.1622			0.8196
No or not Mentioned	351 (37.7)	569 (62.3)		295 (38.5)	452 (61.5)		56 (33.7)	117 (66.3)	
Yes or Borderline	17 (55.3)	20 (44.7)		15 (58.5)	16 (41.5)		2 (38.8)	4 (61.2)	

Hypertension			0.8528			0.7190			0.6795
No	336 (38.4)	539 (61.6)		284 (39.4)	425 (60.6)		52 (33.5)	114 (66.5)	
Yes	32 (37.0)	50 (63.0)		26 (36.6)	43 (63.4)		6 (40.7)	7 (59.3)	

Regular Place of Care			**0.0031**			**0.0300**			**0.0181**
No	45 (58.1)	42 (41.9)		33 (55.4)	32 (44.6)		12 (54.5	10 (45.5)	
Yes, 1 + places	**323 (35.6)**	547 (64.4)		277 (37.03)	436 (62.97)		46 (28.58)	111 (71.42)	

Access to Paid Sick Leave			**<0.0001**			**<0.0001**			**0.0059**
No	245 (46.9)	282(53.1)		200 (47.9)	216(52.1)		45 (42.9)	66 (57.1)	
Yes	123 (27.4)	307(72.6)		110 (28.9)	252(71.1)		13 (18.8)	55 (81.2)	

Alcohol Status			0.1360			0.0958			0.7668
Never	80 (33.0)	156 (67.0)		54 (31.9)	107(68.1)		26 (35.3)	49 (64.7)	
Ever	28 8(40.)	433 (60.0)		256 (41.0)	361(59.0)		32(32.6)	72 (67.4)	

Smoking Status			**0.0192**			**0.0193**			0.9870
Never	244 (35.1)	448 (64.9)		193 (35.4)	344(64.6)		51(33.8)	104 (66.2)	
Ever	124 (46.2)	141(53.8)		117(47.3)	124(52.7)		7 (34.0)	17 (66.0)	

Leisure-Time Physical Activity			**0.0489**			0.0693			0.3656
Inactive/Insufficient	229 (41.5)	354 (58.5)		188(42.6)	275 (57.4)		41 (36.7)	79 (63.3)	
Physically Active	139 (33.4)	235 (66.6)		122(34.3)	193 (65.7)		17 (28.0)	42 (72.0)	

Region of Residence			**0.0497**			0.2435			0.0731
Northeast	44 (32.3)	87 (67.7)		40 (36.0)	65 (64.0)		4 (17.0)	22 (83.0)	
North Central/Midwest	84 (37.2)	138 (62.8)		75 (37.5)	122 (62.5)		9 (33.4)	16 (66.6)	
South	119 (34.8)	224 (65.2)		104 (36.5)	176 (63.5)		15 (25.8)	48 (74.2)	
West	121 (48.5)	140 (51.5)		91 (48.2)	105 (51.8)		30 (49.6)	35 (50.4)	

All p-values are based on chi-square tests for the categorical variables. Bold = Statistical significance. Statistical significance level at p < 0.05.

Note: Individuals born in U.S. territories were included in the non-U.S.-born population.

**Table 2:** Multivariate Logistic Regression Analysis of U.S. Pregnant Women by Demographic, Socioeconomic, and Health Characteristics Association with Prenatal Depression, NHIS 2010–2019.

	Total (N = 957)			U.S.-Born (N = 778)			Non-U.S.-Born (N = 179)		
	AOR	95% CI	P-value	AOR	95% CI	P-value	AOR	95% CI	P-value
Grouped Age									
18–30	0.70	0.48–1.00	0.05	0.61**	**0.41**–**0.91**	**0.01**	1.77	0.64–4.90	0.27
31–44	Ref	–	–	Ref	**–**	–	Ref	–	–

Race									
White	Ref	–	–	Ref	–	–	Ref	–	–
Black	0.57*	**0.33**–**0.98**	**<0.05**	0.53*	**0.30**–**0.96**	**<0.05**	0.54	0.13–2.27	0.40
Other	0.64	0.34–1.22	0.17	0.83	0.36–1.88	0.64	0.35	0.10–1.18	0.08

Grouped BMI									
<18.5–24.9 (Underweight or Normal)	Ref	**–**	**–**	Ref	–	–	Ref	–	–
25–29.9 (Overweight)	1.49	0.95–2.35	0.08	1.60	0.96–2.65	0.06	1.14	0.52–3.82	0.50
≥30 (Obese)	2.08*	**1.31**–**3.30**	**<0.001**	2.18*	**1.33 – 3.59**	**<0.05**	1.46	0.52–4.07	0.47

Household/Family Size									
1–4	Ref	–	–	Ref	–	–	Ref	–	–
≥5	0.87	0.51–1.49	0.61	0.91	0.49–1.67	0.75	0.67	0.20–2.18	

Ratio of Family Income to Poverty Threshold									
<1	1.58	0.97–2.47	0.06	1.58	0.93–2.66	0.08	1.66	0.55–5.01	0.36
≥1	Ref	–	–	Ref	–	–	Ref	–	–

Health Insurance									
No	0.76	0.33–1.74	0.52	0.79	0.28–2.21	0.65	0.22	0.04–1.19	0.07
Yes	Ref	–	–	Ref	–	–	Ref	–	–

Class of Work									
Private	0.64	0.39–1.06	0.08	0.70	0.41–1.19	0.18	0.40	0.10–1.56	0.18
Government	Ref	–	–	Ref	–	–	Ref	–	–

Employment Status									
Employed	Ref	–	–	Ref	**–**	**–**	Ref	**–**	**–**
Unemployed	0.99	0.68–1.43	0.94	1.00	0.67–1.50	0.99	1.31	0.46–3.78	0.61

Diabetes Status									
No or not Mentioned	Ref	–	–	Ref	–	–	Ref	–	–
Yes or Borderline	1.60	0.70–3.68	0.26	1.82	0.72–4.58	0.20	0.89	0.16–5.10	0.89

Hypertension Status									
No	Ref	–	–	Ref	–	–	Ref	–	–
Yes	0.77	0.42–1.41	0.39	0.39	0.38–1.39	0.33	1.62	0.34–7.80	0.54

Access to Paid Sick Leave									
No	2.50***	**1.72**–**3.64**	**<0.001**	2.57***	**1.71**–**3.86**	**<0.001**	1.52	0.45–5.09	0.49
Yes	Ref	–	–	Ref	–	–	Ref	–	–

Regular Place of Care									
No	2.43*	**1.32**–**4.47**	**<0.05**	1.96*	**1.00**–**3.86**	**0.05**	12.54***	**2.92**–**53.79**	**<0.001**
Yes, 1 + Places	Ref	–	–	Ref	–	–	Ref	–	–

Alcohol Consumption							–	–	
Never	Ref	–	–	Ref	–	–	Ref	–	–
Ever	1.40	0.92–2.14	0.11	1.63	0.99–2.67	0.05	0.72	0.30–1.72	0.45

Smoking Status									
Never	Ref	–	–	Ref	–	–	Ref	–	–
Ever	1.24	0.83–1.84	0.29	1.21	0.79–1.85	0.38	1.08	0.27–4.26	0.91

Leisure Time									
Inactive/Insufficient	1.32	0.92–1.90	0.13	1.32	0.89–1.96	0.17	1.83	0.7–4.74	0.20
Physically Active	Ref	–	–	Ref	–	–	Ref	–	–

Region									
Northeast	Ref	**–**	**–**	Ref	–	–	Ref	–	–
North Central/Midwest	0.91	0.52–1.60	0.74	0.84	0.45–1.55	0.57	3.11	0.50–19.51	0.22
South	0.86	0.50–1.48	0.58	0.84	0.46–1.54	0.58	1.92	0.42–9.25	0.38
West	1.83*	**1.04**–**3.20**	**<0.05**	1.67	0.87–3.19	0.12	5.44*	**1.29**–**23.00**	**<0.05**

AOR = Adjusted odds ratio. 95% CI = 95% Confidence interval. Bold = Statistical significance. Statistical significance level at * = p ≤ 0.05, ** = p = 0.01, and *** = p < 0.001. Ref = Reference.

Note: Individuals born in U.S. territories were included in the non-U.S.-born population.

## 3 Results

### 3.1 Prevalence of prenatal depression and statistical difference between prenatal depression and the independent factors among pregnant women

Table 1 estimates the prevalence of prenatal depression among U.S.-born and non-U.S.-born pregnant women. A total sample of 957 pregnant women who participated in the NHIS between 2010 and 2019 in the U.S. were included in this study. A total of 340 (43.7%) U.S.-born and 58 (32.4%) non-U.S.-born pregnant women reported having experienced prenatal depression. Statistically, significant (p < 0.05) difference in the prevalence of prenatal depression in the general sampled population was observed among pregnant women’s race, BMI status, ratio of family income to poverty threshold, regular place of care, access to paid sick leave, smoking status, leisure-time physical activity, and the region of residence. The prevalence of prenatal depression was higher among unemployed U.S.-born pregnant women (42.4%) than their non-U.S.-born counterparts (35.3%). Among the U.S.-born pregnant women race, the highest prevalence of prenatal depression was among individuals identifying as Other race (42.15%), followed by Whites (40.4%) and Blacks (29.3%). The highest prevalence of prenatal depression among non-U.S.-born race was observed among Whites (41.7%). In the total sample, individuals who ever smoked reported a higher prevalence of prenatal depression (46.2%) than those without a smoking history (35.1%). See Table 1 for details.

The between-group comparison showed that U.S.-born individuals with any history of smoking status had a higher prevalence of prenatal depression (47.3%) than non-U.S.-born individuals (34.0%). Also, the highest prevalence of prenatal depression was observed among U.S.-born individuals with a BMI ≥30 (47.1%), followed by 25–29.9 (39.2%), and <18.5 – 24.9 (30.0%). A similar pattern was observed among non-U.S.-born with 42.6%, 32.5%, and 29.6% for a BMI ≥30, 25–29.9, and <18.5 – 24.9, respectively, although there was no statistically significant difference in prenatal depression between these categories (p = 0.51).

The prevalence of prenatal depression among pregnant women without access to paid sick leave was reported to be higher among U.S.-born than non-U.S.-born (47.9% vs. 42.9%). Pregnant women without a regular place of care reported a higher prevalence of prenatal depression among both US-born (55.4%) and non-US-born (54.5) than those with at least one regular place of care [i.e., US-born (37.03%) and non-US-born (28.58%)]. In the total sample, the highest prevalence of prenatal depression by region was reported in the West (48.5%), followed by Midwest/North Central (37.2%), South (34.8%), and Northeast (32.2%). Among non-U.S.-born, the highest prevalence of prenatal depression was reported in the West (49.6%), followed by Midwest/North Central (33.4%), South (25.8%), and Northeast (17.0%) regions of the U.S. While a significant difference in prenatal depression was observed among the combined sample by region of residence, there was no difference among the separate subgroup of U.S.-born and non-U.S.-born. See Table 1 for details.

### 3.2 Association of prenatal depression with independent factors among pregnant women

The multivariable logistic regression analysis is presented in Table 2. The total U.S. pregnant women population without access to paid sick leave reported a higher likelihood of prenatal depression (AOR = 2.50, 95% CI = 1.72–3.64). Similarly, both U.S.-born (AOR = 2.57, 95% CI = 1.71–3.86) and non-U.S.-born (AOR = 1.52, 95% CI = 0.45–5.09) subgroup of pregnant women were more likely to have prenatal depression. However, the finding for non-U.S.-born women was not statistically significant, which could be due to the small sample size for the non-U.S.-born population. Also, in the total sample, U.S. pregnant women without a regular place of care had higher odds of experiencing prenatal depression (OR = 2.43, 95% CI = 1.32–4.47). This was similar for the subgroup of non-U.S.-born pregnant women without a routine place of care (AOR = 12.54, 95% CI = 2.92–53.79). Among the general U.S. pregnant women population, individuals with a BMI ≥30 (AOR = 2.08, 95% CI = 1.31–3.30) were more likely to report prenatal depression compared to underweight/normal-weight individuals (BMI <18.5–24.9). Similarly, among U.S.-born individuals, the likelihood of prenatal depression was higher among obese/overweight (i.e., BMI ≥30) pregnant women (AOR = 2.18, 95% CI = 1.33–3.59) than underweight/normal weight women. The same observation did not apply to non-U.S.-born pregnant women. Further, in the general pregnant women population, individuals residing in the West region of the U.S. were more likely to report prenatal depression (AOR = 1.83, 95% CI = 1.04–3.20). Non-U.S.-born individuals in the Western region of the U.S., compared to those in the Northeastern, also had higher odds of prenatal depression (AOR = 5.44, 95% CI = 1.29–23.00). In the age category, U.S.-born individuals aged 18–30 years (AOR = 0.61, 95% CI = 0.41–0.91) were less likely to experience prenatal depression compared to their counterparts aged 31–44 years.

## 4 Discussion

To the best of our knowledge, this is the first study that utilized the NHIS to investigate the prevalence and factors associated with prenatal depression among U.S.-born and non-U.S.-born pregnant women. As shown in our study, we found several factors associated with prenatal depression among U.S. pregnant population. Factors like age, BMI, access to paid sick leave, regular place of care, and region of residence were found to impact the prenatal depression status of pregnant women. A lack of access to paid sick leave, for instance, may impact prenatal depression among pregnant individuals in the U.S., as they are more likely to experience prenatal depression regardless of their origin of birth. Our results further show that pregnant women who lack a routine place of care had an increased likelihood of reporting prenatal depression. This means they are more likely to miss follow-up for prenatal and depression treatment, which may increase their morbidity and mortality during the postpartum period. Yet, access to paid sick leave improves healthcare access and timely treatment of health conditions, including mental health problems, for pregnant women and ultimately reduces their risk of experiencing postpartum depression. These risk factors for prenatal depression, therefore, could lead to an increased morbidity risk. Wallwiener et al. (2019), for example, investigated mental health disorders during pregnancy and their effect on birth outcomes. The authors observed that women with depression and other mental health diseases had more cesarean sections than those without (Wallwiener et al., 2019).

As shown in this study, a significant majority of pregnant women (i.e., U.S.-born and non-U.S.-born) who did not have a place of routine care had a higher likelihood of reporting being depressed. This finding could also be due to a lack of access to prenatal and postnatal services for these populations. A similar problem is observed nationwide, with young adults and minorities less likely to have a primary care provider (Levine et al., 2020). The current finding further highlights the need to address the lack of a regular place of care problem faced by pregnant women to reduce the likelihood of prenatal depression and other related morbidity and mortality during and after pregnancy. This may ensure that mothers can attend prenatal and postpartum follow-ups and for well women's checkups to enhance their well-being and reduce further health consequences after pregnancy. The findings also show the need for initiatives such as pregnancy medical home and care coordination approaches to effectively aid in improving birth and health outcomes for pregnant women, as shown by some studies (Berrien et al., 2015, Suhag et al., 2017).

The finding that U.S.-born individuals without access to paid sick leave were more likely to experience prenatal depression is concerning. Paid sick leave is essential for pregnant women to access treatment and have improved health. As a result, a lack of access to paid sick leave could delay treatment and make health conditions and pregnancy complications worse. A previous study by Goodman and Schneider found that workers with access to paid sick leave have better sleep quality and less hunger than those without (Goodman and Schneider, 2021). Similarly, another study reported that workers without access to paid sick leave were three times more likely to forgo medical care for themselves and 1.6 times more for their families compared to adult workers with access to paid sick leave (DeRigne et al., 2016). These studies highlight the potential impact of policy actions such as access to paid sick leave on pregnant individuals' health outcomes and that of their families. Delaying care for pregnant women is likely to have more dire consequences, resulting in both prenatal and perinatal depression and other comorbidities such as diabetes and hypertension. For example, a New York City sample study showed that individuals with access to paid sick leave were less likely to seek emergency care and be treated in primary care settings (Ko and Glied, 2021). This finding shows the cost benefits of such an approach in decreasing the potentially expensive use of emergency services when health status has significantly deteriorated among workers without paid leave opportunities.

Moreover, the lack of access to paid sick leave may increase the burden of prenatal depression among pregnant women, as shown in this study, especially if they already have other children under their care. Past studies have revealed that employed parents relied on paid sick leave to care for their children, especially those with chronic conditions (Heymann et al., 1996, Heymann et al., 2010). Consequently, lack of access to paid sick leave for pregnant women might predispose them to health problems such as prenatal depression. Also, this problem is likely to harm children's health as well. A study by Brito et al. found increased activity of high-frequency brain activities among infants of mothers with paid leave (Brito et al., 2022). The finding further highlights the potential health and developmental impact of access to paid sick leave on mother and child health outcomes. Most of the pregnant population does not have access to paid sick leave. The problem of income security and the potential lack of job security for taking extended leave without pay during the prenatal/postpartum period may likely result in depression among mothers in addition to the biological stress of being pregnant. It is imperative to develop strategic approaches to ensure pregnant women can access the care they need before, during, and after pregnancy to maintain quality of life for themselves and their families. Additionally, as Mosley et al. (2021) show, culturally tailored support programs are needed to meet the complex needs of non-U.S.-born mothers and navigate the healthcare system.

Further studies that evaluate the prevalence of prenatal depression among pregnant individuals with access to paid sick leave, type of routine place of care, occupation, and/or industry type by birthplace or citizenship would be vital in gaining a broader understanding of depression among these populations. Such an understanding would aid in devising strategies for follow-up on chronic conditions such as diabetes, postpartum depression and hypertension that often contributes to maternal morbidity and mortality in the prenatal and postpartum period. Moreover, studies focusing on the impact of sociodemographic factors and depression among pregnant women locally could provide a better understanding of effective patient/person-centered medical care and public health collaborative strategies to mitigate the prevalence of prenatal depression and other chronic medical conditions.

We observed differences in the region of residence and prenatal depression. Women residing in the West were more likely to report prenatal depression, whereas those in the South and North Central/Midwest were less likely compared with their counterparts in the Northeast. With a stratified analysis of U.S.-born and non-U.S.-born, only non-U.S.-born living in the West were found significant, with increased odds of prenatal depression compared with those residing in the Northeast. Future research is necessary to examine the regional variations of reported depression and factors contributing to these differences.

## 5 Limitations

Despite the significant contributions of this study, it has some limitations. First, this study uses cross-sectional data; therefore, no causal inferences can be made on the effect of prenatal depression on the independent variables among such a unique population as pregnant women. Second, although we used sampling weight for the analyses, the small samples within some categories likely affected the estimation of prenatal depression among some populations such as the non-U.S.- born. Furthermore, to increase the sample size of our unique study population, we combined three depression questions. Although all screening indicators for depression used in the NHIS are validated, they were self-reported, which may differ from provider-diagnostic validated tools for depression. Also, the employment variable for unemployed includes individuals seeking work and not working for pay. This factor might have impacted the estimation of the results. Finally, social, emotional, and partner support factors were unavailable in the dataset that could have provided further insights into how pregnant women manage prenatal depression. Notwithstanding these limitations, our findings are significant, considering this is a unique sample and not much work has been done with nationally representative datasets.

## 6 Conclusions

We recommend providers use tailored, validated screening tools in care processes for these populations. Given the complexity of health care needs during pregnancy, it is imperative to ensure adequate care coordination with mental health providers. The lack of a regular place of care by the significant number of pregnant populations further shows the decreased likelihood of receiving timely and adequate treatment and follow-ups for conditions like gestational diabetes, preeclampsia, and other maternal and mental health services. Efforts to ensure access to routine care and culturally appropriate mental health services are imperative to addressing depression during pregnancy and postpartum.

## CRediT authorship contribution statement

**Saanie Sulley:** Conceptualization, Methodology, Validation, Visualization, Writing – original draft, Writing – review & editing. **David Adzrago:** Methodology, Validation, Visualization, Writing – original draft, Writing – review & editing. **Lohuwa Mamudu:** Conceptualization, Methodology, Validation, Visualization, Writing – original draft, Writing – review & editing. **Emmanuel A. Odame:** Methodology, Writing – original draft, Writing – review & editing. **Paul H. Atandoh:** Writing – original draft, Writing – review & editing. **Ishmael Tagoe:** Writing – original draft, Writing – review & editing. **David Ruggieri:** Data curation, Formal analysis, Visualization, Writing – review & editing. **Lisa Kahle:** Data curation, Formal analysis, Validation, Visualization, Writing – review & editing. **Faustine Williams:** Project administration, Methodology, Resources, Visualization, Writing – review & editing, Supervision.

## Declaration of Competing Interest

The authors declare that they have no known competing financial interests or personal relationships that could have appeared to influence the work reported in this paper.

## Data Availability

The datasets generated and/or analyzed during the current study are available in the National Health Interview Survey repository, https://www.cdc.gov/nchs/nhis/data-questionnaires-documentation.htm.
